# Appreciative Friends

**Published:** 1889-02

**Authors:** 


### APPRECIATIVE FRIENDS.

In our December issue this request was made: Let each of our present subscribers resolve to write *one paper* for our pages, and also to secure *one new* subscriber for 1889; before the end of the year he will feel himself amply repaid for his labor.

The responses to this request have been numerous; much more general than we had dared to hope for. To all of these friends we return our thanks, both for their new subscribers and for their promised manuscripts.

Amongst the responses to our request one is unique, both for its kindly expressions of interest and secondly for the *practical* work done in our behalf. The writer, Dr. W. J. Martin, of Pittsburgh, says: "I presume you will wonder what you ever did to me that I should send you such a list of new subscribers as the inclosed! I will tell you. I have been reading The Homœopathic Physician ever since its first number was published; I would like every homœopathic physician to be a reader of it for the good I feel sure it would do them. * * * Last evening at the regular monthly meeting of the Allegheny County Homœopathic Medical Society, I took occasion to call the attention of the members to the character of The Homœopathic Physician, as being the truest homœopathic journal in the land, and read to them the important announcement concerning the Repertory of Characteristics to be published as a supplement to the journal. Then, after a few closing remarks, I called upon every one, not already a subscriber, to give me his subscription. The result is the fifteen new names and thirty dollars inclosed."

The editors of this journal desire to return thanks to Dr. Martin for his letter and for the new subscribers; he is a man after our own heart! May many more of our friends "go and do likewise."—Editors.

